# Predictors of caregiver burden among primary caregivers of cancer patients at Hawassa oncology center Northern, Ethiopia, 2022: institution-based cross-sectional study

**DOI:** 10.1186/s12875-023-02170-x

**Published:** 2023-10-28

**Authors:** Eman Ali, Yacob Abraham, Tinbete Samuel, Aklile Tsega, Mastewal Aschale

**Affiliations:** Faculty of Health Sciences, College of Medicine and Health Sciences, Hawassa University, Hawassa, Ethiopia

**Keywords:** Cancer, Caregivers, Caregiver’s burden, Psychological distress

## Abstract

**Background:**

Approximately 60,960 people are diagnosed with cancer each year, and more than 44,000 people die from it. Family caregivers face a range of difficulties because cancer affects many facets of life, such as nursing care, communication, financial issues, and emotional conflicts. Consequently, family caregivers are more susceptible to developing demanding physical and mental conditions. Despite these problems, cancer remains the most neglected and underfunded health problem in Ethiopia. Therefore, this study aimed to assess the caregiver burden experienced by family caregivers of patients with cancer; as well as its associated factors.

**Methods:**

An institutional-based cross-sectional study was employed among 347 family caregivers of cancer patients who attended Hawassa University Comprehensive Specialized Hospital Oncology Center from May 30 to July 30, 2022. The data were checked for completeness and consistency and then coded. The coded data were entered into Epi-data version 4.6 and then exported into Statistical Package for Social Science (SPSS) version 25 for analysis. The caregiver’s burden was assessed by a short form of Zarit burden Interview. The explanatory variables, like clinical and care-related factors, were assessed by a structured questionnaire. Family caregivers’ perceptions of social support were assessed by the multidimensional scale of perceived social support. Binary logistic regression was used to assess the strength of the association between outcome and explanatory variables. Each explanatory variable was entered separately in the bivariate analysis, and a variable with a p-value less than 0.25 goes further for multivariate analysis to control the possible confounding. The statistical significance of the factors influencing the outcome variable was declared in multivariate logistic regression analysis using an adjusted odds ratio at a 95% confidence interval when a p-value < 0.05.

**Results:**

The response rate of the caregiver was 100%. This study reported that 66.6% (95% CI 61.5–71.5) of the caregivers had a high caregiver burden. Being female, caring hours, previous history of hospitalization, and sleeping hours were significantly associated with the caregiver’s burden.

**Conclusion:**

In this finding, more than two-thirds of the caregivers had a higher caregiver burden. This suggested that there is a need to focus on and give more attention to caregivers to decrease their burden by including caregiver burden in routine nursing activities by the oncology unit, and further study should be done at the national level using other study designs.

## Background

Cancer is a major cause of illness and mortality in the world. It is characterized by abnormal cell proliferation and spreads through the movement of cells through the blood and lymphatic systems to other parts of the body. Cancer cells, unlike normal cells, do not undergo apoptosis; as they continue to grow, proliferate, and disseminate [1]. Over 50 million cancer deaths occur annually worldwide, and of these, 80% occur in developing countries [2].

According to Hoffman and Mitchell, the burden of caregiving for a chronic disease “results from a process encompassing numerous connected elements, including socioeconomic features, caregiver resources, and stressors” [3]. Family caregivers offer long-term care for cancer patients but often receive little training, information, or support to perform this important duty [4]. The diagnosis of cancer and the changes that occur as a result of the reality of care can often disorganize the family system and necessitate changes to accommodate the treatment demands in everyday life [5]. Caregivers’ burden is the result of the physical, social, economic, and psychological effects of providing care on their lives as seen by them [6].

Family caregivers confront a range of difficulties since cancer affects so many facets of life, such as nursing care, communication, money issues, and emotional conflicts [7]. As a result, family caregivers are more susceptible to developing several demanding physical and mental conditions [5]. Typically, caregivers are beset by psychological problems like anxiety, hopelessness, and loneliness [8].

In 2020, there were around 10 million cancer deaths and 19.3 million new cases worldwide [9]. Based on GLOBACAN (2020), there were 700,000 cancer deaths and 1.1 million new cases on the African continent [9]. Due to population increase, aging, and the prevalence of cancer, it is predicted that in the next 20 years, the incidence will double [10]. The caregiver burden experienced a mild to moderate level of burden at 71.6% [11]. The magnitude of the caregiver burden was reported at 47.4% in Malaysia [12], 37.5% in India [13], 46.19% in Nigeria [6], and 10.6% in Ethiopia [11]. According to data from Ethiopia’s national cancer control program, cancer causes 5.8% of all fatalities in the country. Approximately 60,960 people are diagnosed with cancer each year, and more than 44,000 people die from it. There is limited research in Africa including our country Ethiopia on cology centers on the burden among primary caregivers of cancer patients and it is the most neglected and underfunded health problem in the country [14]. Health professionals still remain unaware of the fact that patients and caregivers have an interdependent relationship therefore fail to address the needs of caregivers as a part of the therapeutic strategy when they provide care for the patients. As a result, the study aimed to assess the caregiver burden experienced by cancer patients as well as its associated factors. With the rising number of cancer cases and caregiver burdens around the world, it’s more important than ever to examine the caring components and identify solutions to improve the well-being of family caregivers.

## Methods and materials

### Study area, period, and design

An institutional-based cross-sectional study was conducted from May 30 to July 30, 2022, at Hawassa University Comprehensive Specialized Hospital Oncology Center. It is far, 275 km, from Addis Ababa, the capital city of the country. HUCSH has various units such as a dermatology clinic, laboratory unit, pathology unit, psychiatry clinic, ENT clinic, physiotherapy unit, surgical unit, and internal medicine with a sub-specialty of cardiology and neurology, ophthalmology, radiology, oncology, and others to serve the community. HUCSH Oncology center, there were a total of 780 and 63 new patients joining per year and month, respectively. Approximately 240 patients visit at OPD, and 150 patients are followed up per month. In the cancer center, there were a total of 15 nurses, 2 general practitioners, and 2 senior oncologists.

### Study Population

All family caregivers of diagnosed cancer patients who were attending HUCSH oncology center were the source populations. All family caregivers of patients diagnosed with cancer who were on treatment, follow-up, or care at the time of data collection in the oncology center were the study population.

### Inclusion and exclusion criteria

All primary caregivers of a cancer patient who had a treatment or care follow-up at the HUCSH oncology center and received care for more than two weeks were included [4, 15, 16]. Professional caregivers or paid caregivers and primary caregivers under 18 years of age were excluded.

### Sample size determination and procedure

The sample size was determined by using a single proportional formula under the following assumptions; a proportion of 71.6% [11] from the previous study, a margin of error of 0.05, and a 95% confidence interval.

$$n = \frac{{{{(za/2)}^2}p{\rm{ }}\left( {1 - p} \right)}}{{{d^2}}},n = \frac{{{{{\rm{(1}}{\rm{.96)}}}^2}{\rm{[0}}{\rm{.71(1 - 0}}{\rm{.71)}}}}{{{{(0.05)}^2}}} = 316$$

After adding a 10% non-response rate for the participants the final sample size was 347.

On the oncology unit’s registration book there was lists of cancer patients who received treatment or underwent follow-up. Using the patient list we calculate the caregiver sampling interval. There were 780 patients in HUCSHS oncology center for consecutive two months. Then the sampling interval (K) was determined using the formula as follows; N/n = 780/347, which gives a sampling interval of approximately 2. Then the data was collected from each study participant (caregivers) with an interval of two until the desired sample size was reached. In all cases, K was 2 and 1 was selected randomly and the 1st caregiver was taken as a first sample and then taking every two caregiver until the required sample size is obtained.

### Data collection tool

The data were collected in Amharic, the national language of the country, through a semi-structured, pre-tested, and interviewer-administered questionnaire by four nursing professionals. The questionnaires have four parts. The first part included a structured, closed-ended questionnaire to measure the socio-demographic information of the study participants. The second part included the clinical and care-related factors that were assessed by a structured questionnaire. The third part included the outcome variable, which was assessed by a short form of Zarit Burden Interview (ZBI) [17]. It has 12-item questionnaires, and they are defined subjectively. The questions were centered on the caregiver’s emotional responses. Each question is graded on a 4-point Likert scale, from never to almost always present. The total score ranges from 0 to 48. The fourth part included perceived social support that the family caregivers receive from friends, family and significant other was measured using MSPSS, it is a 12-item instrument used to measure perceived social support with 7-likert scale. The Scale has three subscales measuring support from significant other, family and friend each having four items [18].

### Data quality measure

A pre-test was done one week before the actual data collection at Adare Hospital on 5% of the sample size with a Cronbach’s alpha of 0.75) to test the quality and effectiveness of the questionnaires on the study subjects. The training was given to data collectors. The data collection supervision was undertaken by the principal investigator. The completeness of the data was handled and stored properly.

### Data processing and analysis

The data were checked for completeness and consistency and then coded. The coded data were entered into Epi-data and then exported into Statistical Package for Social Science (SPSS) version 25 for analysis. Descriptive statistics (frequencies, tables, graphs, percentages, and means) were used to characterize study subjects. Binary logistic regression was used to assess the strength of the association between outcome and explanatory variables. In the bivariate analysis, each explanatory variable was entered separately, and a variable with a p-value less than 0.25 advanced to the multivariate analysis to control for potential confounding. The statistical significance of the factors influencing the outcome variable was declared in multivariate logistic regression analysis using an adjusted odds ratio at a 95% confidence interval (CI) when a p-value < 0.05.

### Operational definition

#### Family caregivers

The terms family caregiver and informal caregiver refer to an unpaid family member, friend, or neighbor who provides care to an individual who has an acute or chronic condition and needs assistance to manage a variety of tasks, from bathing, dressing, and taking medications to tube feeding and ventilator care [19].

#### Caregiver’s burden

high caregiver burden when the caregiver scores > 10 and low caregiver burden when the caregiver scores < 10 from zerit questionnaire [17].

## Results

### Socio-demographic characteristics of caregivers

Three hundred forty-seven participants were included in the current study with a 100% response rate. Of the total participants more than half (56.8%) were males and more than half of (53.8%) the participants were from arban areas. Among the study participants (11.2%) were cared by their sisters. Of the total respondents nearly two-third of ( 63.7%) of the participants were married and employed. The mean age of the respondent was 26.45 years with an SD of 9.2. Among those, more than one-third (35.2%) were found in the age group of 20–34 years followed by 35–49 years (Table 1).

**Table 1:** Socio-demographic characteristics of primary caregivers of cancer patients at HUCSH oncology center Ethiopia, in 2022 (n = 347)

Socio demographic characteristics of the caregiver		Frequency	Percent
Sex of caregiver	Male	197	56.8%
	Female	150	46.2%
Residency of caregiver	Urban	186	53.8%
	Rural	161	46.4%
Relationship to the patient	Spouse	78	22.5%
	Son	76	21.9%
	Daughter	60	17.3%
	Sister	39	11.2%
	Brother	32	9.2%
	Mother	29	8.4%
	Father	29	8.4%
	Others	4	1.2%
Marital status	Single	96	27.7%
	Married	221	63.7%
	Divorced	30	8.6%
Occupation	Employed	219	63.1%
	Unemployed	128	36.9%
Education level	Illiterate	14	4%
	Primary	138	39.8%
	Secondary	104	30.1%
	Higher level	91	26.2%
Religion	Muslim	111	32%
	Orthodox	125	36%
	Protestant	104	30%
	Others	7	2%
Age [20]	<20 years	36	10.4%
	20–34 years	122	35.2%
	35–49 years	116	34%
	>50 years	71	20.5%
Monthly income (ETB) [11]	<3000	183	52.7%
	3001–7000	112	32.3%
	7001-10,000	38	11%
	>10,000	14	4%
Number of family	0–3 individuals	111	32%
	4–7 individuals	183	52.7
	>8 individuals	53	15.3%

ETB = Ethiopian Birr

### Socio-demographic characteristics of the patients

Of the total participants, more than two-third (66.9%) were females. Nearly half of the patients (48.4%) were Urban areas. More than half of the patients came from rural areas. Regarding their occupation, 212 (61.1%) of the patients were employed. Nearly one-third (32.6%) of the patient was educated upto primary level (Table 2).

**Table 2:** Socio-demographic characteristics of the patient at HUCSH Oncology Cancer Center Ethiopia, in 2022 (n = 347)

Variables	Categories	Frequency	Percent
Sex	Male	115	33.1%
	Female	232	66.9%
Residency	Urban	168	48.4%
	Rural	179	51.6%
Marital status	Single	81	23.3%
	Married	231	66.6%
	Widow	17	4.9%
	Others	18	5.2%
Occupation	Employed	212	61.1%
	Unemployed	136	38.9%
Education level	Illiterate	94	27.1%
	Primary	113	32.6%
	Secondary	77	22.2%
	Higher level	63	18.2%
Religion	Muslim	109	31.4%
	Orthodox	125	36%
	Protestant	98	28.2%
	Others	15	9.3%

ETB; Ethiopian Birr

### Clinical and care-related characteristics

Of the total cancer patients, (44.4%) were found stage three of cancer diseases. More than one-fourth (28.5%) of the patients were diagnosed with breast cancer followed by gastrointestinal cancer (25.1%). Among these, (79.6%) of the patients were receiving chemotherapy and more than two-third of the patients have no comorbidity (Table 3).

**Table 3:** Frequency distribution of clinical and care-related characteristics of the caregivers and patients at HUCSH oncology center Ethiopia, in 2022 (n = 347)

Variables	Categories	Frequency	Percent
stage of cancer	Stage 1	18	5.2%
	Stage 2	42	12.1%
	Stage 3	154	44.4%
	Stage 4	133	38.3%
Types of cancer	Breast cancer	99	28.2%
	Cervical cancer	35	10.1%
	Gastrointestinal CA	87	25.1%
	HCC	22	6.3%
	Lung cancer	39	11.2%
	NHL	34	9.8%
	Pancreatic cancer	17	4.9%
	Others	14	4%
Treatment	Chemotherapy	277	79.6%
	Combination	70	20.2%
Comorbidity	Yes	70	20.2%
	No	277	79.6%
Physical symptoms	No physical symptoms	108	31.1%
	Pain	145	41.8%
	Loss of appetite	44	12.7%
	Others	50	14.4%
Do you know the diagnosis disease	Yes	329	94.8%
	No	18	5.2%
Are you living with your relative	Yes	253	72.9%
	No	94	27.1%
Previous history of hospitalization	Yes	249	71.8%
	No	98	28.2%
Caring hour	1–3	31	8.9%
	3–5	54	15.6%
	5–8	124	36.7%
	> 8	138	39.8%
Sleeping hour	< 7	258	68.6%
	> 7	109	31.4%

HCC: Hepatocellular Carcinoma; NHL: Non-Hodgkin Lymphoma

### Perceived social support

**Fig. 1:**
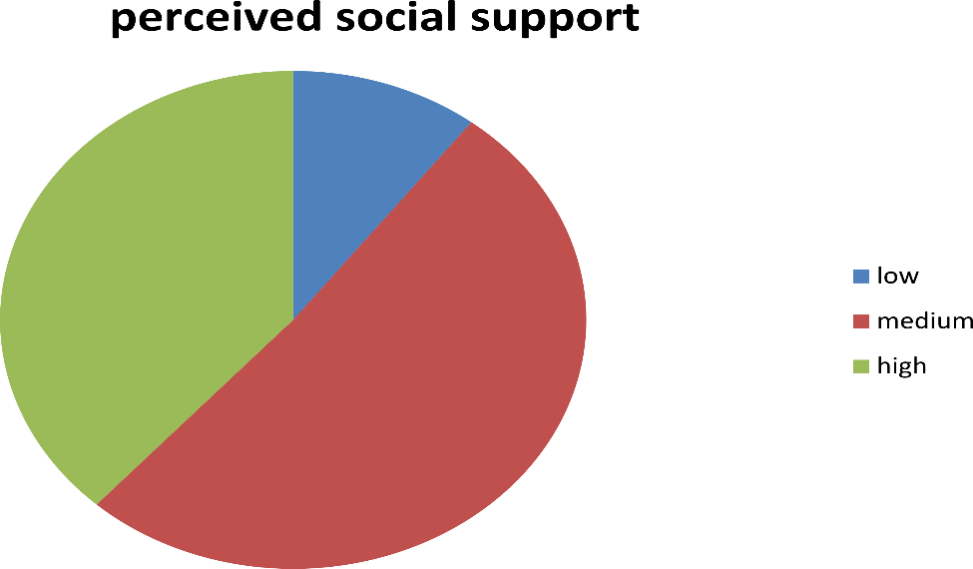
Perceived social support among primary caregivers of cancer patients at HUCSH cancer center Hawassa Ethiopia 2022 (n = 347)

The perceived social support that the family caregivers receive from friends, family and significant other was measured using MSPSS, it is a 12-item instrument used to measure perceived social support with 7-likert scale. The Scale has three subscales measuring support from significant other, family and friend each having four items. The total mean score was 22.79 with standard deviation of 6.04.the by using the scale response descriptors as a guide .In this approach any mean scale ranging from 1 to 2.9 could be considered as low support, score of 3 to 5 could be considered moderate support, a score from 5.1 to 7 could be considered high support. In the current study 36 (10.4%) of the care givers have low social support and 178 (51.3%) of the caregivers have moderate social support.133 (38.3%) of the caregivers have high social support (Fig. 1).

### Caregivers burden

Caregivers burden is measured by using the short form Zarit burden interview scale which has 12 items each having 4 likert scale. It has a total scoring of 0 up to 48 and a person who get a total score of less than 10 has a lower caregivers burden and a caregiver score greater than 10 has a greater caregivers burden This study revealed that 66.6% of the caregivers had a high caregiver burden, while 33.4% of the caregivers had a low caregiver burden.

### Factors associated with the caregiver’s burden

While performing the bivariate logistic regression analysis, sex of the patient, stage of cancer, physical symptoms, previous history of hospitalization, duration of care, and sleeping hours were the candidates for further analysis in the multivariate logistic regression analysis. In the final model of multivariate logistic regression analysis, sex of the patient, previous history of hospitalization, sleeping hour, and duration of care were found to be statistically significant with the caregiver’s burden.

The odds of developing a caregiver’s burden increase two times in female patients as compared to male patients (AOR = 2.00 95% CI :1.13–3.53). The odds of developing caregiver burden were two times higher in caregivers with a previous history of the hospitalized patient as compared to caregivers who had no history of the hospitalized patient (AOR = 1.99;95% CI 1.12–3.52). In this study, caregivers who spent greater than eight hours for caregiving are three times more likely to have a caregiver burden than caregivers who gave care for less than eight hours (AOR = 3.40;95% CI: 1.30,8.93). The odds of developing caregiver burden were two times higher in caregivers who slept less than seven hours as compared to caregivers who slept greater than seven hours (AOR = 2.36;95%CI 1.28–4.35) (Table 4).

**Table 4:** Bivariate and multivariate logistic regressions of caregiver’s burden among family caregivers of cancer patients at HUCSH oncology center, in 2022 (n = 347)

Variables		Caregivers burden		COR (95% CI)	AOR (95% CI)	p-value
		High	Low			
Sex of patient	**Female**	**166**	**66**	1.93(1.214–3.084) *	2.00(1.13–3.53) **	**0.017**
	Male	65	50	1	1	
Caring hour	3–5 h.	37	17	2.64(1.06–5.57)	2.71 (0.96–7.64)	0.060
	5–8 h.	100	24	5.06(2.19–11.67)	0.96 (0.3-,2.47)	0.956
	**> 8 h.**	**80**	**58**	1.68(0.76–3.66) *	3.40 (1.30–8.93) **	**0.013**
	1–3 h.	14	17	1	1	
Previous history of Hospitalization	**Yes**	**179**	**70**	2.26(1.40–3.57) *	1.99(1.12–3.52) **	**0.019**
	No	52	46	1	1	
Sleeping hour	**< 7 h**	**159**	**67**	1.62(1.02–2.56) *	2.36 (1.28–4.35) **	**0.006**
	> 7 h	72	49	1	1	
Physical symptom	Pain	103	42	1.96(1.16–3.31)	0.47(0.21–1.11)	0.084
	Loss of appetite	32	12	2.13(0.99–4.58)	0.92(0.39–2.13)	0.837
	Others*	36	14	2.06(1.00-4.25)	1.00(0.36–2.77)	0.998
	No physical symptoms	60	48	1	1	
Stage of cancer	Stage 2	34	8	1.63(0.45–5.92)	2.14(0.53–8.54)	0.283
	Stage 3	79	75	0.41(0.14, 1.19)	0.49(0.15, 1.56)	0.225
	Stage 4	105	28	1.44(0.47–4.39)	1.36(0.41–4.50)	0.616
	Stage 1	13	5	1	1	

*=Vomiting, fatigue, weight loss; AOR = Adjusted Odds Ratio; COR = Crude Odds Ratio; CI = Confidence Interval;1 = Reference group; *p-value < 0.25; **p-value < 0.05

## Discussion

Even though giving care by family caregiver for ill patient in Ethiopia is an usual norm; they face different problems during providing a care for patients like physical, psychological, social, and financial problem. The current study showed that the majority of caregivers 231(66.6%) had high caregivers’ burden whereas 116 (33.4%) of the caregivers have low caregivers’ burden with a total mean of 16.65.

The result of the current study showed that the magnitude of caregiver burden among the study participants was 66.6% at 95% CI: 61.5–71.5. In the final model of multivariate logistic regression analysis, being female, sleeping hours, duration of caregiving, and prior history of hospitalization were statistically significantly factors with the caregiver’s burden.

In this study, the level of caregiver burden (66.6%) was lower than in a study conducted at Jimma Hospital’s oncology unit among primary caregivers of cancer patients, which was (71.6%) [11]. The possible reason for the discrepancy might be due to methodological differences that is the previous study was used a long-form of Zarit burden interview with 22 items but, the current study used a short-form of Zarit burden interview with 12 items. Additionally, the previous study used caregiver burden inventory (CBI) and moreover it might be differences in sampling technique.

However, the current study finding is higher than a study done in two areas of Malaysia; Sarawak and Selango [12, 21] reported that the caregiver burden was 55.6%, and 47.4% respectively and South Korea showed (48.1%) of caregivers experience a caregiver burden [22] and Iraq [23]. The possible rationale for the above difference might be due sociocultural and economic differences which can directly affect the caregiver’s burden in different nations.

The results of the current study revealed that being female, care patients greater than eight hours per day, sleeping hours, and previous history of hospitalization were statistically significant factors in the final model of multivariate logistic regression model with the outcome variable.

Being female can increase the caregiver’s burden by two times as compared to the male patients (AOR = 2.00, P = 0.017). This is contradicted by a study in Iran stating that male patients had more burden on the caregivers than female patients [24]. The reason for the discrepancy might be that one-quarter of the caregivers (25.2%) was the spouses of the patients. When the wife suffers from certain types of cancer, the entire family, especially the husband, might be confronted with the new situation. As a result, he may experience increased responsibilities by default, increasing his burden. The difference might be due to cultural differences in different nations in which in the Ethiopian context females are more prone to responsibilities both at home and in the community but in the Iranian culture, a man has more active roles in society, in comparison with a woman.

Giving care for a long period of time had higher caregiver burden compared from caregivers who care less hour within a day (AOR = 3.40, p = 0.013). This finding was supported by a study done in Nigeria [6] and Korea [25]. Other studies done in Nigeria among caregivers of breast cancer patients support the current finding [26]. The current study contradicts a study conducted in Pakistan, which showed that a shorter duration of caregiving was associated with a higher burden [27]. This might be due to the fact that longer caregivers engage in caregiving activities and they are more burden due to financial constraints and the lack of time to perform their daily routine. The loss of social support over a longer period of time during caregiving may also be another rationale [26]. On the other hand, incongruence between study findings might be due to cultural variation in the study across different nations. In the current study, a sleeping hour is significantly associated with the caregiver’s burden. Caregivers who sleep less than seven hours within a day are (AOR = 2.35, P = 0.006**)** times more likely to be burdened than caregivers who sleep greater than seven hours. This might be due to caregivers who sleep less being more susceptible to stress and diseases related to sleep deprivation, which can increase the caregiver’s burden. This result is in line with a study conducted in China that revealed the caregiving burden was independently associated with poor sleep quality [28]. **T**he present study findings showed that a previous history of hospitalization is significantly associated with a higher caregiver burden with an (AOR = 1.99, P = 0.019). This is consistent with study done in [26] As the disease advances, the patient begins to exhibit numerous symptoms and medication-related side effects that may necessitate hospitalization. This rationale may be explained by the patient’s stage of cancer since the majority of patients are at stages three and four. During the treatment period, the family caregiver is expected to provide both financial and emotional support. As a result, the process of the patient being hospitalized increases the caregiver’s burden.

### Strength and limitation

The study’s attention has been drawn to a group of people who have been neglected and disadvantaged people about whom insufficient research has been done. Perceived social support was assessed as a variable that can influence caregiver’s burden which is not used in the previous study. Additional strength of the study was an adequate sample was taken. The limitation of the study was that, even though the survey tried to access the caregiver burden among primary caregivers of cancer patients in the oncology unit, it was difficult to generalize the finding to another group of the population. Another limitation of the study was that it was a cross-sectional study, so it did not demonstrate a causal effect.

## Conclusion

In this finding, more than two-thirds of the caregivers had a higher caregiver burden. This suggests that there is a need to focus and give more attention to caregivers to decrease their burden by including caregiver burden in routine nursing activities by the Hawassa University oncology unit, especially for those who are females, caring for a long hour, previous history of hospitalization, and small sleeping hours. Further study should be done at the national level using other study designs.

## Data Availability

The raw data may be available upon reasonable request by the corresponding author.
